# INTERNALIZED HOMOPHOBIA MEDIATING THE RELATIONSHIP OF SELF-CONCEALMENT ON DEPRESSION IN LGB OLDER ADULTS

**DOI:** 10.1093/geroni/igac059.3036

**Published:** 2022-12-20

**Authors:** Christopher Calzada, Carl St Goar, Matthew Hollander, Vennisia Mo, Jamie Kiefer, Ben Hougaard, Nicole Carre, Rowena Gomez

**Affiliations:** Palo Alto University, Palo Alto, California, United States; Palo Alto University, Palo Alto, California, United States; Palo Alto University, Palo Alto, California, United States; Palo Alto University, Palo Alto, California, United States; Palo Alto University, Palo Alto, California, United States; Palo Alto University, Palo Alto, California, United States; Palo Alto University, Palo Alto, California, United States; Palo Alto University, Palo Alto, California, United States

## Abstract

Previous research has demonstrated that self-concealment of sexual orientation may negatively affect the wellbeing of lesbian, gay, and bisexual (LGB) adults (Hu, Wang, & Wu, 2013). Additionally, studies have indicated that internalized homophobia significantly predicts depressive symptoms in LGB older adults (Sharma & Subramanyam, 2020). The present study examined how internalized homophobia may mediate the predictive relationship between self-concealment on depression symptoms in LGB older adults. As part of a larger study, participants (Nf301) responded to several questions and measures including the Sexual Orientation Self-Concealment Scale, The Internalized Homophobia Scale, and the Center for Epidemiological Studies Depression Scale. Using the Baron and Kenny’s Method for Mediation (1986), the data analysis indicated that internalized homophobia fully mediates the relationship between self-concealment and depression severity (ps < .05). These results suggest that older adults who self-conceal their sexual orientation may experience an increase in depressive symptoms because of internalized homophobias. These findings may help clinicians better understand the mechanisms contributing to the high rates of depressive symptoms within the older LGB population. Furthermore, the findings suggest that cognitive restructuring of thoughts related to internalized homophobia and self-concealment may improve depressive severity in older LGB adults.

